# Solitär fibröser Tumor in einem follikulären Adenom der Schilddrüse

**DOI:** 10.1007/s00292-024-01353-2

**Published:** 2024-09-12

**Authors:** Sarah Theurer, Simon Schimmack, Hideo A. Baba

**Affiliations:** 1 Institut für Pathologie, Universitätsklinikum Essen, Universität Duisburg-Essen, Hufelandstr. 55, 45147 Essen, Deutschland; 2 Klinik für Allgemein- und Viszeralchirurgie, Bethesda-Krankenhaus Duisburg, Duisburg, Deutschland

**Keywords:** Tumor in Tumor Schilddrüse, Schilddrüsentumor, Spindelzelliger Tumor der Schilddrüse, NAB2-STAT6-Fusion, Metastase solitär fibröser Tumor, Tumor-in-Tumor thyroid, Thyroid tumor, Spindle cell tumor of the thyroid, NAB2-STAT6 fusion, Metastasis solitary fibrous tumor

## Abstract

Bei einer 28-jährigen Patientin lag ein größenprogredienter Knoten der Schilddrüse vor, der aufgrund einer lokalen Beschwerdesymptomatik operativ entfernt wurde. Histologisch konnte ein solitär fibröser Tumor (SFT) in einem Schilddrüsenadenom diagnostiziert werden. Molekulargenetisch lag eine spezifische *NAB2-STAT6*-Fusion vor.

Solitär Fibröse Tumoren kommen selten in der Schilddrüse vor und sind dort sowohl als Primär- wie auch als Sekundärmanifestation beschrieben. Dabei erfolgen die Diagnostik, die Prognoseabschätzung und die Therapieentscheidung analog zu SFT anderer Lokalisation.

**Zusatzmaterial online:**

Zusätzliche Informationen sind in der Online-Version dieses Artikels (10.1007/s00292-024-01353-2) enthalten.

## Abstract (en)

A 28-year-old female patient developed a 5.5 cm progressive thyroid nodule that was surgically removed due to local symptoms. Histologically, a solitary fibrous tumor (SFT) in a thyroid adenoma was diagnosed. The tumor had a specific NAB2-STAT6 fusion.

Solitary fibrous tumors rarely occur in the thyroid gland and are described as both primary and secondary manifestations. The diagnosis, prognosis assessment and treatment decision are carried out analogously to SFT of other localizations.

## Anamnese

Eine 28 Jahre alte Patientin stellte sich in der Schilddrüsensprechstunde mit einem größenprogredienten rechtsseitigen Schilddrüsenknoten und lokaler Beschwerdesymptomatik vor. Auf eine präoperative Funktionsszintigraphie oder Feinnadelaspiration wurde aufgrund der Beschwerdesymptomatik verzichtet. Es wurde die klinische Indikation zur Hemithyreoidektomie rechts gestellt, welche komplikationsfrei durchgeführt werden konnte.

## Befund

*Makroskopisch* lag ein 23 g schweres und 5,5 × 4,5 × 4,5 cm großes rechtsseitiges Hemithyreoidektomiepräparat vor. Die Schnittfläche wurde nahezu komplett von einem polyzystischen, rötlichen Knoten eingenommen. Eine prominente Kapsel war nicht auffällig. Makroskopisch ergaben sich keine Malignitätshinweise.

**Abb. 1:**
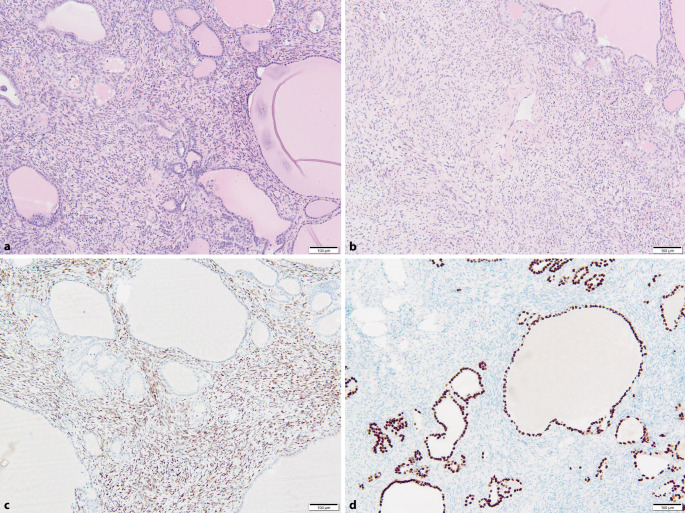
Histologisch stellt sich ein spindelzelliges Proliferat dar, das zwischen den Follikeln eines Schilddrüsenknotens wächst (**a**, HE-Färbung, Vergr. 100:1). Im spindeligen Tumoranteil zeigen sich Gefäßneubildungen, die angedeutet auch Verzweigungen ausbilden (**b**, HE-Färbung, Vergr. 100:1). Während die spindeligen Tumorzellen STAT6 nukleär kräftig exprimieren (**c**, Immunhistochemie STAT6, Vergr. 100:1), bleiben die Zellen der follikulären Strukturen negativ. Diese sind allerdings positiv angefärbt für den thyreoidalen Marker TTF1 (**d**, Immunhistochemie TTF1, Vergr. 100:1), während die Spindelzellen nicht reagieren

*Histologisch *stellte sich nur noch im Randbereich etwas druckatrophes, follikulär gebautes Schilddrüsenparenchym dar (Abb. 1). Das Bild wurde überwiegend geprägt von einem Knoten mit einerseits größenvarianten, kolloidgefüllten Schilddrüsenfollikeln mit Resorptionsvakuolen als Zeichen der endokrinen Aktivität. Andererseits zeigte sich zwischen den Follikeln des Knotens ein prominentes spindelzelliges Proliferat mit Gefäßneubildungen. Die Spindelzellen hatten einen länglichen Kern und ein blass eosinophiles, lang ausgezogenes Zytoplasma. Pro 10 HPF lag 1 Mitose vor. Sonstige Atypien oder Nekrosen kamen im Tumor nicht vor. Die Gefäße zeigten eine angedeutete Verzweigung.

Der gesamte Tumor war durch eine schmale Bindegewebskapsel begrenzt, in der sich weder Gefäßeinbrüche noch Kapseldurchbrüche darstellen ließen.

*Immunhistochemisch* färbten sich die follikulären Strukturen des Knotens für thyreoidale Marker (Thyreoglobulin, TTF1, PAX8) an, während das spindelzellige Proliferat für diese Marker negativ war. Beide Komponenten waren negativ für CD31, während die tumoreigenen Gefäße mit diesem Marker deutlich hervorgehoben wurden. CD34 markierte die Gefäße, war aber auch in einigen der Spindelzellen schwach positiv exprimiert. In der Färbung mit dem Antikörper gegen STAT6 waren die Spindelzellen nukleär kräftig angefärbt. Die proliferative Aktivität in den spindeligen Tumorzellen lag bei der Färbung mit Ki67 bei 1 %.

*Molekulargenetisch* konnte mittels Genfusionsanalyse (Archer) eine *NAB2*-Exon4-*STAT6*-Exon-2-Fusion nachgewiesen werden.

## Diagnose

Aufgrund der Morphologie, des passenden immunhistochemischen Profils und des Nachweises einer spezifischen Genfusion stellten wir die Diagnose eines solitär fibrösen Tumors (SFT) in einem follikulären Adenom der Schilddrüse. Ob es sich dabei um die Metastase eines SFTs anderer Lokalisation handelt oder um einen in loco entstandenen Primarius, konnte histologisch nicht zuverlässig beantwortet werden.

Zur Einschätzung des individuellen Risikos der Patientin wurde der Demicco-Score ermittelt [1]. Dabei ergab sich für die Konstellation Patientenalter < 55 Jahre (= 0 Punkte), Tumorgröße 5–10 cm (= 1 Punkt) und 1 Mitose/10 HPF (= 1 Punkt) ein Gesamtpunktwert von 2, welches einem Low-risk-Tumor entspricht (vgl. Supplement Tab. 1).

## Therapie und Verlauf

Der postoperative Verlauf der Patientin gestaltete sich komplikationslos. Für das weitere Prozedere wurde der Fall in einer interdisziplinären Tumorkonferenz besprochen. Ein daraufhin angefertigtes Ganzkörper-FAPI(Fibroblasten-Aktivierungs-Protein)-PET-CT zeigte keine Hinweise auf weitere Manifestationen des SFTs. Es kann daher davon ausgegangen werden, dass es sich bei dem Herd in der Schilddrüse um den Primärtumor handelt. Eine weiterreichende Therapie war daher nicht notwendig. Eine regelmäßige Nachüberwachung in jährlichen Abständen mit sonographischer Kontrolle der Halsregion wurde empfohlen.

## Diskussion

Spindelzellige Läsionen der Schilddrüse kommen häufig vor. Das diagnostische Spektrum reicht dabei von reaktiven Veränderungen in nodulär follikulären Schilddrüsenerkrankungen (ehemals Struma nodosa [2]) über entzündliche Veränderungen bis hin zu benignen und malignen schilddrüseneigenen Tumoren. Mesenchymale spindelzellige Tumoren der Schilddrüse sind insgesamt selten, vereinen aber unterschiedlichste Entitäten. So sind beispielsweise Schwannome [3] und glattmuskuläre Tumoren [4] als Einzelfälle beschrieben. Das primäre Angiosarkom der Schilddrüse ist der häufigste Vertreter maligner mesenchymaler Tumoren der Schilddrüse [5]. Eine Übersicht über die häufigsten spindelzelligen Läsionen der Schilddrüse gibt Tab. 1. Differentialdiagnostisch muss in jedem Fall auch an eine Metastase eines spindelzelligen Tumors anderer Lokalisation in der Schilddrüse gedacht werden.

**Tab. 1:** Übersicht über die wichtigsten spindelzelligen Läsionen in der Schilddrüse und deren Haupt-Diagnosemerkmale

Reaktiv/entzündliche Veränderungen	Benigne	Reaktive Veränderungen in follikulär nodulärer Erkrankung (ehemals Struma nodosa)	Multinodöse Schilddrüse mit reaktiven Veränderungen (frische und alte Einblutungen/resorptive Veränderungen/Cholesterinkristalleinlagerungen/Verkalkungen)
	Benigne	Reaktive Veränderungen bei Z. n. Feinnadelaspiration	Klinisch Z. n. Feinnadelpunktion; Granulationsgewebe/Narbengewebe; residuelle Einblutungen
	Benigne	Fibröse Variante der Autoimmunthyreopathie Hashimoto	Merkmale einer Hashimoto-Erkrankung; Destruktion der Schilddrüsenfollikel mit extensiver Fibrose; keine zytologischen Atypien; residuelle, kolloidentleerte Follikelinseln mit Plattenepithelmetaplasie typisch
	Benigne	Fibrosierende Thyreoiditis (Riedel-Thyreoiditis)/IgG4-assoziierte Thyreoiditis	Keine zytologischen Atypien; Schilddrüsenfollikel destruiert und atroph; lymphozytäres Beiinfiltrat mit Plasmazellen und eosinophilen Granulozyten; begleitende obliterierende Phlebitis; vermehrte Ansammlungen IgG4 positiver Plasmazellen; pseudoinfiltratives Wachstum der Fibrose über die Organgrenze hinaus
Tumoren mit Follikelepithelursprung	Benigne	Spindelzelliges Adenom	Immunhistochemische Expression von Schilddrüsenmarkern; Ausschluss von Malignitätskriterien (Kernveränderungen des PTC, Gefäßeinbrüche, invasives Wachstum)
	Maligne	PTC, spindelzelliger Subtyp	Immunhistochemische Expression von Schilddrüsenmarkern; Kernmerkmale des papillären Schilddrüsenkarzinoms zumindest in Abschnitten des Tumors; meistens liegen andere Subtypen des PTC zusätzlich vor. Abgrenzung zum ATC über blande Kernmorphologie und wenige/fehlende Mitosen
		FTC, spindelzelliger Subtyp	Immunhistochemische Expression von Schilddrüsenmarkern; Gefäßeinbrüche und/oder Kapseldurchbrüche; Abgrenzung zum ATC über blande Kernmorphologie und wenige/fehlende Mitosen
		ATC, sarkomatoider Subtyp	Immunhistochemische Expression der Schilddrüsenmarker deutlich abgeschwächt bzw. nicht vorhanden; Nekrosen, (atypische) Mitosen, Kernpleomorphie häufig vorhanden; Kombination mit epitheloiden Anteilen häufig
Tumoren mit C‑Zell-Ursprung	Maligne	MTC, spindelzelliger Subtyp	Immunhistochemische Expression von Chromogranin und Calcitonin
Spindeliger epithelialer Tumor mit Thymus-ähnlicher Differenzierung (SETTLE)	Maligne	–	Biphasischer Tumor mit spindeligen Tumorzellen, die zwischen glandulären Strukturen liegen; immunhistochemische Positivität für CK7 in beiden Tumoranteilen; immunhistochemische Negativität für thyreoidale Marker, Calcitonin, S100, CD5
Mesenchymale Tumoren	Benigne	Schwannom	Wechselnde Zellularität (Antoni-A/Antoni-B-Muster); nukleäre Pallisadierung; immunhistochemische Expression von S100
	Benigne	Leiomyom	Immunhistochemische Positivität für Aktin/Desmin
	Benigne/maligne	Solitär fibröser Tumor (SFT)	Immunhistochemische Positivität für STAT6; spezifische Fusion *NAB2-STAT6*
	Maligne	MPNST	Wechselnde Zellularität; evtl. Nekrosen; fleckige immunhistochemische Positivität für S100
	Maligne	Leiomyosarkom	Immunhistochemische Positivität für Aktin/Desmin
	Maligne	Angiosarkom	Immunhistochemische Positivität für CD 31/CD34/ERG
Metastatische Absiedlungen	Maligne	Malignes Melanom	Immunhistochemische Positivität für Melan A/S100/HMB45/SOX10

*PTC* papilläres Schilddrüsenkarzinom, *FTC* follikuläres Schilddrüsenkarzinom, *ATC* anaplastisches Schilddrüsenkarzinom, *MTC* medulläres Schilddrüsenkarzinom, *MPNST* maligner peripherer Nervenscheidentumor

Besonders herausfordernd bei der Interpretation sämtlicher Läsionen in der Schilddrüse ist das Auftreten von intranodal gelegenen Proliferaten, die follikulär sein können, aber auch jede andere Morphologie ausbilden können. Dabei sollten in erster Linie reaktive/regressive Phänomene bedacht werden. Als Prädilektionsort der Entstehung anderer Tumoren sollten intranodal gelegene Proliferate im Zweifel immer immunhistochemisch untersucht werden, um die Expression thyreoidaler Marker zu bestätigen bzw. auszuschließen. Benigne Tumoren ohne Follikelzellursprung (z. B. Schwannome) entstehen in der Schilddrüse bevorzugt in bereits vorbestehenden Knoten (Tumor-in-Tumor-Phänomen) [6]. Auch in dem vorliegenden Fall war das Spindelzellproliferat innerhalb eines follikulären Knotens zwischen den schilddrüseneigenen Follikeln gelegen. Auch an die Metastase eines malignen Tumors anderer Lokalisation sollte gedacht werden. Dabei sind wiederum besonders präexistente Schilddrüsenknoten ein Hauptentstehungsort für Tumorabsiedlungen.

Solitär fibröse Tumoren sind fibroblastäre Tumoren mit intermediärem malignen Potenzial, die zumeist (2/3 der Fälle) an der Pleura und pulmonal vorkommen. Fallbeschreibungen von SFT anderer Lokalisation existieren jedoch aus nahezu allen extrapulmonalen und extrathorakalen Organen [7]. Solitär fibröse Tumoren der Schilddrüse sind in der Literatur bislang 48-mal beschrieben [1]. Die Diagnose kann, wie in allen anderen Lokalisationen, anhand einer spezifischen *NAB2-STAT6*-Fusion, die zu einer immunhistochemisch nachweisbaren Expression von STAT6 im Zellkern führt, eindeutig und zuverlässig gestellt werden. Es kommen sowohl primäre SFT als auch Metastasen von SFT anderer Lokalisation in der Schilddrüse vor. In aller Regel verhalten sich primäre SFT der Schilddrüse benigne mit einem indolenten klinischen Verlauf. Nur Einzelfälle wurden als maligne mit später auftretenden Metastasen beschrieben [8]. Der klinische Verlauf hängt unter anderem von der *STAT6-NAB2*-Fusionsvariante ab [9]. Unabhängig vom genetischen Hintergrund haben Demicco et al. [1] zur Vorhersage der Wahrscheinlichkeit einer späteren Metastasierung einen Risikoscore entwickelt, der die Tumoren anhand der Parameter Patientenalter, Tumorgröße, Mitoseanzahl und Nekrosen in „low risk“, „intermediate risk“ und „high risk“ unterteilt (vgl. Supplement Tab. 1).

Bei SFT der Schilddrüse handelt es sich fast immer um Zufallsbefunde, da keine spezifischen Merkmale in präoperativen bildgebenden Verfahren existieren. In aller Regel sind daher SFT der Schilddrüse bereits primär chirurgisch komplett exzidiert und benötigen keinerlei Nachtherapie. Eine Staginguntersuchung mittels spezifischer FAPI-PET-CT zur Darstellung etwaiger Metastasen oder eines Primarius anderer Lokalisation wird empfohlen [10]. Ebenso eine langfristige Nachsorge, da SFT anderer Lokalisationen auch nach längerer Zeit (> 5 Jahre) noch Metastasen ausbilden können [11]. Für maligne SFT der Schilddrüse existiert aufgrund der absoluten Rarität der Diagnose bislang keine gängige Handlungsempfehlung. Eine Einzelfallentscheidung zu Therapie und Nachsorge über interdisziplinäre Tumorboards ist daher notwendig [12].

## Fazit für die Praxis

- Solitär fibröse Tumoren (SFT) können in allen Organen des Körpers als Metastase oder Primarius auftauchen und kommen auch in der Schilddrüse vor. Der Demicco-Score sollte zur Prognoseabschätzung bestimmt werden.
- Bei spindelzelligen Proliferaten in der Schilddrüse muss differentialdiagnostisch ein SFT bedacht werden. Die Diagnose ist dank spezifischer Genfusionen und einer wegweisenden Immunhistochemie eindeutig zu stellen.
- Besondere diagnostische Aufmerksamkeit muss auf intranodal gelegene nichtfollikuläre Proliferate in der Schilddrüse gelegt werden, da Schilddrüsenknoten selber ein Prädilektionsort für andere Tumoren sein können (Tumor-in-Tumor-Phänomen).

## Supplementary Information

**Supplement Tab. 1. **Risikostratifizierung durch den Demicco-Score allgemein und bezogen auf den hier thematisierten Fall [1].
