# Supplementary material for: Solitary fibrous tumor in a thyroid follicular adenoma
Source: Pathologie (Heidelb). 2024 Sep 12;45(6):404–8. [Article in German] doi: 10.1007/s00292-024-01353-2 (PMC11511758; doi:10.1007/s00292-024-01353-2)
Supplement: Supplementary file 1 — Supplement Tab. 1. Risikostratifizierung durch den Demicco-Score allgemein und bezogen auf den hier thematisierten Fall [1]. [file 292_2024_1353_MOESM1_ESM.docx]

**Supplement Tabelle 1.** Risikostratifizierung durch den Demicco-Score allgemein und bezogen auf den hier thematisierten Fall [1].

| **Risikofaktor** | **Punktwert** | **Vorliegender Fall** |
| --- | --- | --- |
| Alter |  |  |
| <55 Jahre | 0 | 0 |
| ≥55 Jahre | 1 |  |
| Tumorgröße |  |  |
| <5 cm | 0 |  |
| 5 bis <10 cm | 1 | 1 |
| >10 cm | 2 |  |
| Mitoseanzahl / 10HPF |  |  |
| 0 | 0 |  |
| 1-3 | 1 | 1 |
| ≥4 | 2 |  |
| Bewertung (Gesamtpunktzahl) | 0-2 = low risk  3-4 = intermediate risk  5-6 = high risk | 2 = low risk |
